# Increased Feeding Speed Is Associated with Higher Subsequent Sympathetic Activity in Dogs

**DOI:** 10.1371/journal.pone.0142899

**Published:** 2015-11-16

**Authors:** Nobuyo Ohtani, Yuta Okamoto, Kanako Tateishi, Hidehiko Uchiyama, Mitsuaki Ohta

**Affiliations:** 1 Laboratory of Effective Animals for Human Health, School of Veterinary Medicine, Azabu University, 1-17-71 Fuchinobe, Chuo-ku, Sagamihara, Kanagawa, Japan; 2 Laboratory of Animal Facilitated Therapy, Faculty of Agriculture, Tokyo University of Agriculture, 1737 Funako, Atsugi, Kanagawa, Japan; Institut Pluridisciplinaire Hubert Curien, FRANCE

## Abstract

Although the domestication process has altered the feeding behavior of dogs, some breeds still demonstrate a remarkable ability to gorge, and will eat exceptionally large quantities of food whenever it is available. Lesions in the ventromedial hypothalamus increase appetite and lead to obesity, suggesting that the autonomic nervous system plays an important role in feeding. Focusing on the autonomic activities closely involved in food intake, we investigated sympathetic activities before and after feeding in dogs. The subjects were 56 healthy dogs of 21 different breeds (29 males and 27 females). Based on feeding habits, the 56 dogs were divided into three groups: Fast (n = 19), Slow (n = 24) and Leftover (n = 13). The feeding speed and the amount of food per mouthful of the Fast dogs were significantly greater than those of the Slow and the Leftover dogs. The plasma norepinephrine level in dogs of the Fast group was significantly increased after feeding, while those in the Slow and Leftover groups were significantly decreased after feeding, compared with the pre-feeding concentrations. The low frequency/high frequency ratio of heart rate variability is a good indicator of sympathetic activity and was also significantly higher in the Fast group than in the other groups. Delayed feeding using automatic feeding equipment decreased the plasma norepinephrine concentration and low frequency/high frequency ratio observed after feeding in dogs of the Fast group. In conclusion, dogs eating rapidly with less chewing, which indicates increased sympathetic activity during feeding, may benefit from delayed feeding. The slow eating may activate the parasympathetic nervous system after feeding, which could enhance the activity of the digestive system.

## Introduction

Animals in which the ventromedial hypothalamus has been destroyed have increased appetites and exhibit obesity, which reduces the activity of the sympathetic nervous system [1,2,3] and enhances that of the parasympathetic nervous system [4,5], respectively. Fasting for 48 hours suppresses the sympathetic nervous system activity [6], while overeating activates the sympathetic nervous system [7,8]. Thus, the autonomic nervous system plays an important role in feeding. Bray (2000) [9] has emphasized the importance of the sympathetic nervous system in food intake, suggesting that a high sympathetic tone is a potential measure of satiety in humans and animals, and that the activation of the sympathetic nervous system is a satiety system [10,11]. The increase in the sympathetic activity is associated with a decrease in food intake [9].

Dogs, *Canis familiaris*, are classified as carnivores, but many breeds have become omnivorous during domestication [12,13]. Although domestication has altered their feeding behavior, some breeds still demonstrate a remarkable ability to gorge, and will eat exceptionally large quantities of food whenever it is available [14,15]. In addition, dogs, who can freely consume food, exhibit obesity [15,16,17]. Several dog breeds have reputations for being able to consume large meals very rapidly, and this may be a result of competitive feeding in their ancestors, the wolves [15,18]. It appears that the satiety centers in these dogs are not functioning. They experience difficulty during training when switching from food to a secondary reinforcement, and thus they sometimes reject the owner’s commands when a food reward is not used [19]. While food is a convenient reinforcement tool, it is difficult to build good owner–dog relationship if the owners do not understand their dog’s levels of interest in food [19]. Okamoto et al. [19] also demonstrated that the feeding speed (g/sec) and the amount of food consumed per mouthful did not depend on sex, age, or the dogs’ body size. At present, few studies have been conducted to evaluate what factors effect canine feeding behavior through the above mentioned mechanisms.

We investigated how the activity of the sympathetic nervous system relates to food intake in dogs, to understand why some dogs continue to eat while others do not.

In this study we chose the plasma concentration of norepinephrine (NE) and the heart rate variability (HRV) as measures of the experimental outcomes. NE is synthesized and stored within the peripheral sympathetic nerve endings. In response to nerve impulses NE is released from nerve endings to stimulate the effector cells within a microenvironment [6]. However, some NE is released into the blood. HRV represents the beat-to-beat variation in heart rate generated by the interplay of sympathetic and parasympathetic nerve activities. The spectral analysis of HRV is divided into two major oscillatory components [20], the high-frequency (HF) domain (0.15–1.00 Hz), which reflects parasympathetic activity, and the low-frequency (LF) domain (0.04–0.15 Hz), which reflects both the sympathetic and parasympathetic activity. Therefore, it is possible to evaluate each domain by dividing it into sympathetic and parasympathetic components using a spectral analysis [21].

## Materials and Methods

### Ethics

All of the procedures were approved by the Animal Experiments Ethics Committee of Azabu University in accordance with the World Medical Association Declaration of Helsinki (Approval# 120806).

### Subjects

The subjects were 56 healthy dogs of 21 different breeds (29 males and 27 females), except brachycephalic dog breeds. The research was conducted at two sites, with 34 dogs studied at the World Ranch in Osaka, Japan and 22 dogs studied at the Murase Dogs Training Center in Kanagawa, Japan (Table 1). They were born and raised in the same facilities, which are regulated by the Japanese law, the Law of Humane Treatment and Management of Animals. All of the dogs were sexually intact and 12–96 months of age (51.4 ± 3.8 months, excepting one dog whose age was unknown). They were housed in individual metal cages (90 cm in width and 180 cm in height) and provided with commercial dog food (Adult Maintenance, Nutro Products Inc., Victorville, CA, USA), according to the dietary recommendation, which was based on the body weight, age, and activity level of each dog. We also considered the body composition scale (Body Condition System at Nestle PURINA) [22] and rated the dogs’ body condition in five of nine grades based on the following conditions: ribs palpable without an excess fat covering, waist observed behind ribs when viewed from above, and abdomen tucked up when viewed from the side. The dogs were taken for walks daily in the morning (9:00 to 11:00 am) and evening (15:00 to 17:00 pm), and were inspected at least once daily to monitor their health and well-being. A veterinarian and facility staff members checked several health conditions: appetite, thirst, defecation, urination, behavior, lethargy, and so forth. The dogs had been taught basic commands, such as “sit” and “down”, by the staff at each facility using the positive reinforcement technique [23,24].

**Table 1:** Experimental groupings of dogs based on feeding habits.

Groups	n (male, female)	Age (month)	Weight (kg)
Fast	19 (13, 6)	48.4±6.0	28.3±2.6
Slow	24 (12, 12)	55.2±6.4	27.9±3.6
Leftover	13 (4, 9)	48.5±7.4	22.2±4.0

Fast: Dogs that consumed their food completely and did not chew every mouthful more than once

Slow: Dogs that consumed their food completely and chewed every mouthful more than once

Leftover: Dogs that partially consumed their food

### Classification of the dogs according to their feeding pattern

**Fig 1:**
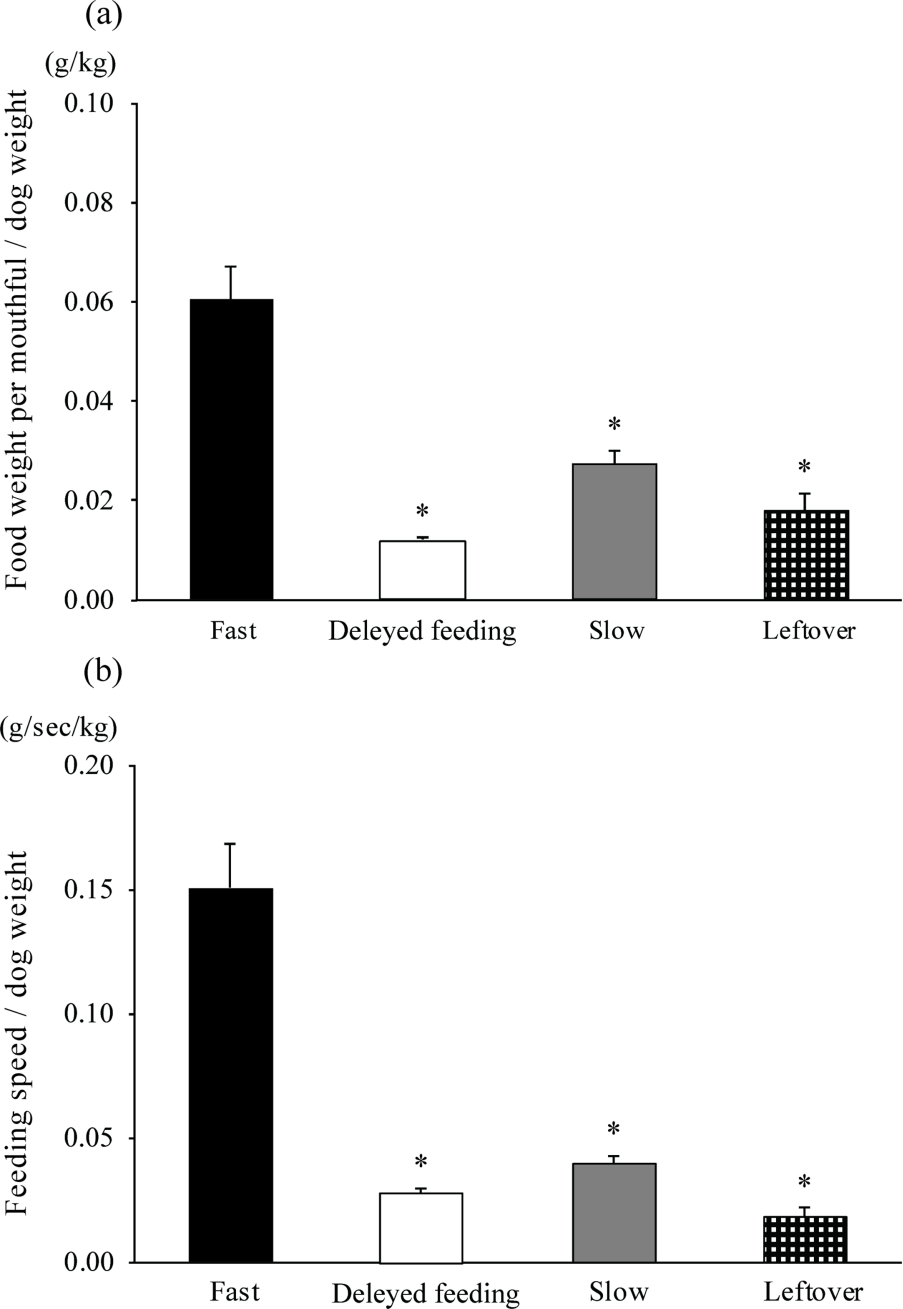
(a) The amount of food per mouthful per body weight (g/kg), and (b) the feeding speed per body weight (b/sec/kg) for each dog in the three feeding groups, Fast, Slow, and Leftover, and the delayed feeding subjects in the Fast group. The F(3,61) values are 16.94 and 10.91 for the upper (a) and lower (b), respectively. Fast, Slow, and Leftover were the same as in Table 1. Fast with delayed feeding was the subgroup of Fast dogs fed using automatic feeding equipment. The differences among groups and between pre- and post-feeding in each group were analyzed using a one-way factorial ANOVA and Dunnett’s test. * significantly lower compared with the Fast group (*p* < 0.01).

To classify the dogs, we recorded their normal feeding behavior for 10 min using a digital video camera (GSC- R60, Toshiba, Tokyo, Japan). The experimental meal sizes in this study were determined by factors such as the body condition scale, daily activity, age, and body weight. After measuring the food weight and feeding duration, the feeding speed (g/sec) and the amount of food consumed per mouthful per body weight (g/kg) were calculated. The latter is the total grams of food available to the dog divided by the number of mouthfuls taken. Based on the results, the dogs were divided into two groups, those who consumed their food completely and those who only partially consumed their food (Leftover). The dogs who consumed their food completely were then divided into two sub-groups based on whether they chewed every mouthful more than once (Slow) or not (Fast). Thus, the dogs were divided into three final groups based on their feeding pattern, Leftover, Slow or Fast (Table 1, Fig 1A and 1B).

### Delayed feeding

**Fig 2:**
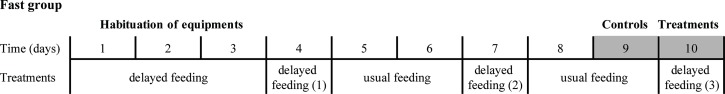
A schematic diagram of the delayed-feeding schedule for the Fast group, which were dogs that consumed their food completely and did not chew every mouthful more than once. Habituation to the delayed automatic feeding equipment occurred over three consecutive days prior to examining the effects of delayed feeding. Blood samples for the comparison between the controlled and delayed-feeding conditions were taken from different dogs. Controls (n = 10) were Fast dogs with delayed feeding, and their blood samples were collected before (Pre) and after (Post) usual feeding on the 9^th^ day of the delayed-feeding experiment. On the 10^th^ day of the experiment, pre- and post- delayed-feeding blood samples were collected (Treatments, n = 9).

The average time that it took to finish eating the food in the Slow group was 285 ± 26 sec. Using the delayed automatic feeding equipment (Sharper Image Corp., Walnut Creek, CA, USA), it took almost the same time for dogs in the Fast group to eat. After adjusting this equipment, each individual was able to eat more than the appropriate amount of food in ~280 seconds once. Nineteen dogs of the Fast group (Table 1) were used as the treatment dogs for delayed feeding and were acclimated to the feeding device twice a day between 09:00 and 10:00 and between 17:00 and 18:00, when they were usually fed, for three consecutive days. Thereafter, the experimental procedure of delayed feeding was performed, as shown in Fig 2.

### Blood sampling

Blood samples were taken from different dogs in the Fast group for a comparison between controlled and delayed feeding conditions. Nine dogs of the Fast group with delayed feeding (Treatments in Fig 2) were randomly selected for blood sampling, and they were transferred to individual cages one hour before feeding and were maintained at rest for 30–40 min. Controls (n = 10) were Fast dogs with delayed feeding and their blood samples were collected before (Pre) and after (Post) usual feeding on the 9^th^ day of the delayed-feeding experiment. On the 10^th^ day of the experiment, pre- and post- delayed-feeding blood samples were collected as treatments (n = 9) (Fig 2). Blood samples for the measurement of catecholamines were collected in EDTA-coated tubes from the cephalic veins. The samples for pre-feeding were from the right cephalic vein and those for post-feeding were from the left. Samples of 2–3 mL volume were kept on ice and the plasma was separated, which was stored at –80°C until measurement.

### Heart rate variability (HRV)

A Polar^®^ Ra PS800CX digital HR monitor and a Polar^®^ Wearlink strap with a transmitter (Polar^®^ Electro Öy, Kempele, Finland) were used to measure the inter-beat (RR) intervals. The Wearlink straps with two electrodes were adapted for use in dogs as previously reported [25]. The data were filtered using Kubios HRV software, version 2.0 (The Biosignal Analysis and Medical Imaging Group at the Department of Physics, University of Kuopio, Kuopio, Finland), and the power spectrum for the frequencies was calculated. The components of the LF (low frequency, 0.04–0.15 Hz) to HF (high frequency, 0.15–1.00 Hz) ratio (LF/HF), reflects the parasympathetic and sympathetic activities, respectively. The experimental numbers of the Fast group with and without delayed feeding were 9 and 10, respectively.

### Assay

Plasma NE was analyzed using a reversed-phase HPLC system with an electrochemical detector (ECD-300, EICOM, Osaka, Japan). The carbon electrode was held at 0.45 V. The column was used to measure NA with a 2.1 mmφx 150 mm reversed-phase column (EICOMPAK CA-50DS, EICOM) and a 3 mmφx 4 mm guard column (PREPAKSET-CA, EICOM). The mobile phase consisted of 0.1 M disodium phosphate buffer containing 1 mL/L EDTA-2Na (50 mg/mL), 1000 mg/L sodium 1-octanesulfonate (ion-pair chromatography reagent, Nacalai Tesque Inc., Kyoto, Japan) and 10% methanol at pH 6.0.

### Statistical analyses

A correlation between the amount of food per mouthful per body weight (g/kg) and the feeding speed per body weight (g/sec/kg) was calculated using the Spearman rank correlation coefficient. The differences among groups, and between pre- and post-feeding in each group, were analyzed with a one-way factorial ANOVA and Dunnett’s test, and a paired t-test, respectively. The differences in plasma norepinephrine (NE) concentrations between the Fast group’s control and delayed feeding dogs were compared using an unequal variance test (Welch’s test) because of the unequal sample sizes. The statistical values for data are included in the figure legends. Results are expressed as means ± SE. *P* values < 0.05 or better were considered significant.

## Results

**Fig 3:**
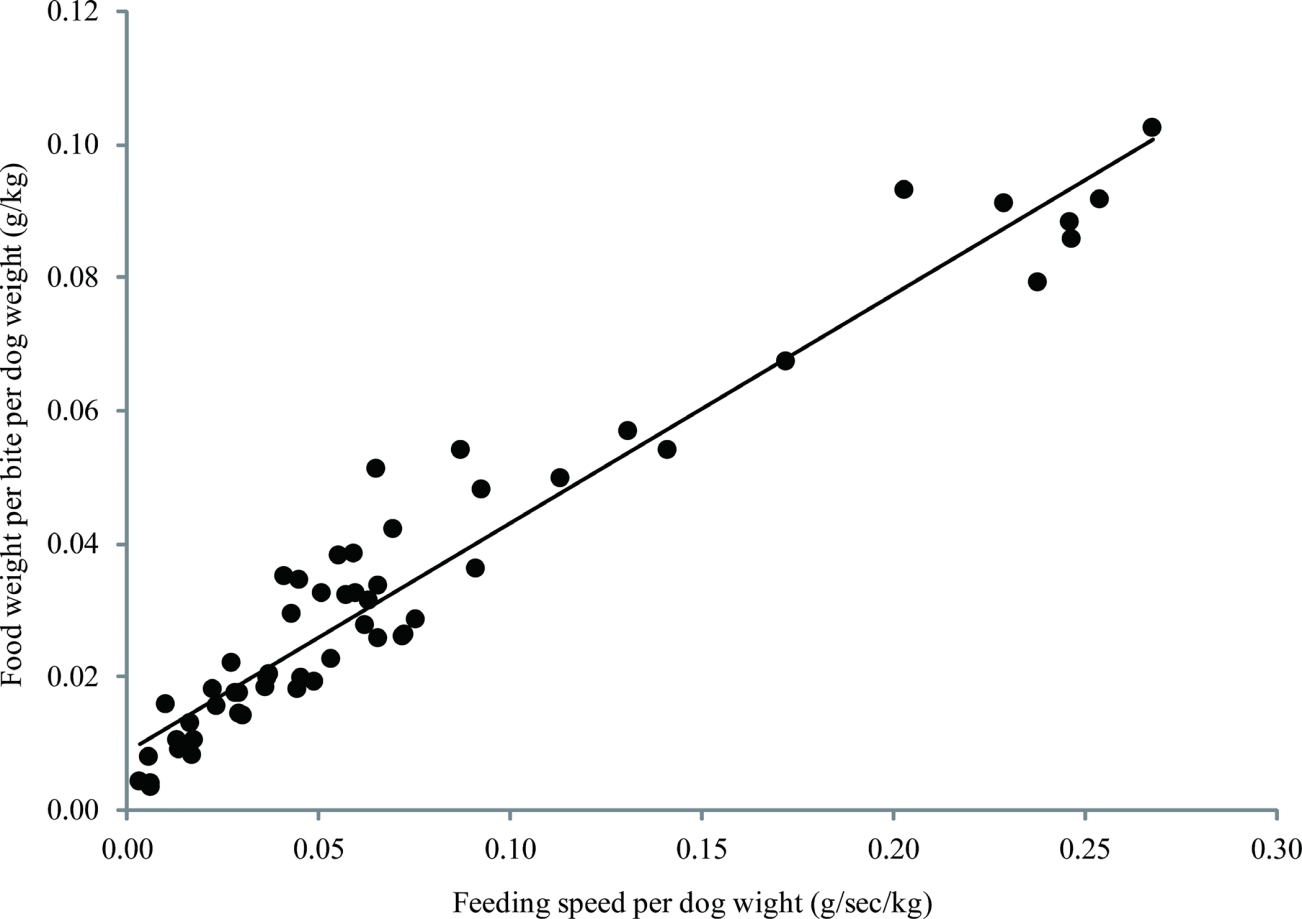
A correlation between the amount of food per mouthful per body weight (g/kg) and the feeding speed per body weight (g/sec/kg). A significant positive correlation between the two was observed (rs = 0.97, *p* < 0.01).

According to feeding patterns, the 56 dogs were divided into three groups: Fast (n = 19), Slow (n = 24) and Leftover (n = 13). A significant positive correlation between the feeding speed and the amount of food per mouthful per body weight for each dog is shown in Fig 3 (Spearman rank correlation coefficient: rs = 0.97, *p* < 0.01). Additionally, the feeding speed and the amount of food per mouthful per body weight of the Fast group were significantly faster and greater, respectively, than those of the Slow and the Leftover groups (one-way factorial ANOVA and Dunnett’s test, Fig 1A and 1B).

The plasma NE concentrations in dogs of the Fast group were significantly increased post-feeding, while those in the Slow and Leftover groups were significantly decreased after feeding, compared with the pre-feeding values (paired t-test, Table 2). The delayed-feeding subjects of the Fast group significantly decreased their plasma NE concentrations post-feeding (one-way factorial ANOVA and Dunnett’s test, Table 2).

**Table 2:** Plasma norepinepherine levels pre- and post-feeding in dogs divided into three groups, Fast, Slow and Leftover, and the subgroup, Fast with delayed feeding, based on feeding habits.

Groups	n	Norepinephrine (pg/ml)			
		pre	post	t-value	changes (post-pre)
Fast	19	371.5 ± 17.5	514.7 ± 44.4 ^a^ ^,^ ^x^	-4.18	143.2 ± 34.2 ^y^
Slow	24	320.1 ± 19.6	275.4 ± 19.4 ^b^	3.09	-44.7 ± 14.5
Leftover	13	314.0 ± 33.2	206.7 ± 23.5 ^b^	4.67	-107.2 ± 23.0
Fast with delayed feeding	9	391.4 ± 20.4	329.9 ± 20.6 ^b^	4.89	-61.7 ± 12.6

Mean ± SE

Fast, Slow and Leftover: See Table 1.

Fast with delayed feeding: Subgroup of Fast dogs fed with automatic feeding equipment.

a) significantly increased, compared with the pre-value (paired t-test, p < 0.05)

b) significantly decreased, compared with the pre-value (paired t-test, p < 0.05)

x) significantly higher, compared with the other groups [F(3, 61) = 18.54, p < 0.01]

y) significantly higher, compared with the other groups [F(3, 61) = 20.66, p < 0.01]

**Fig 4:**
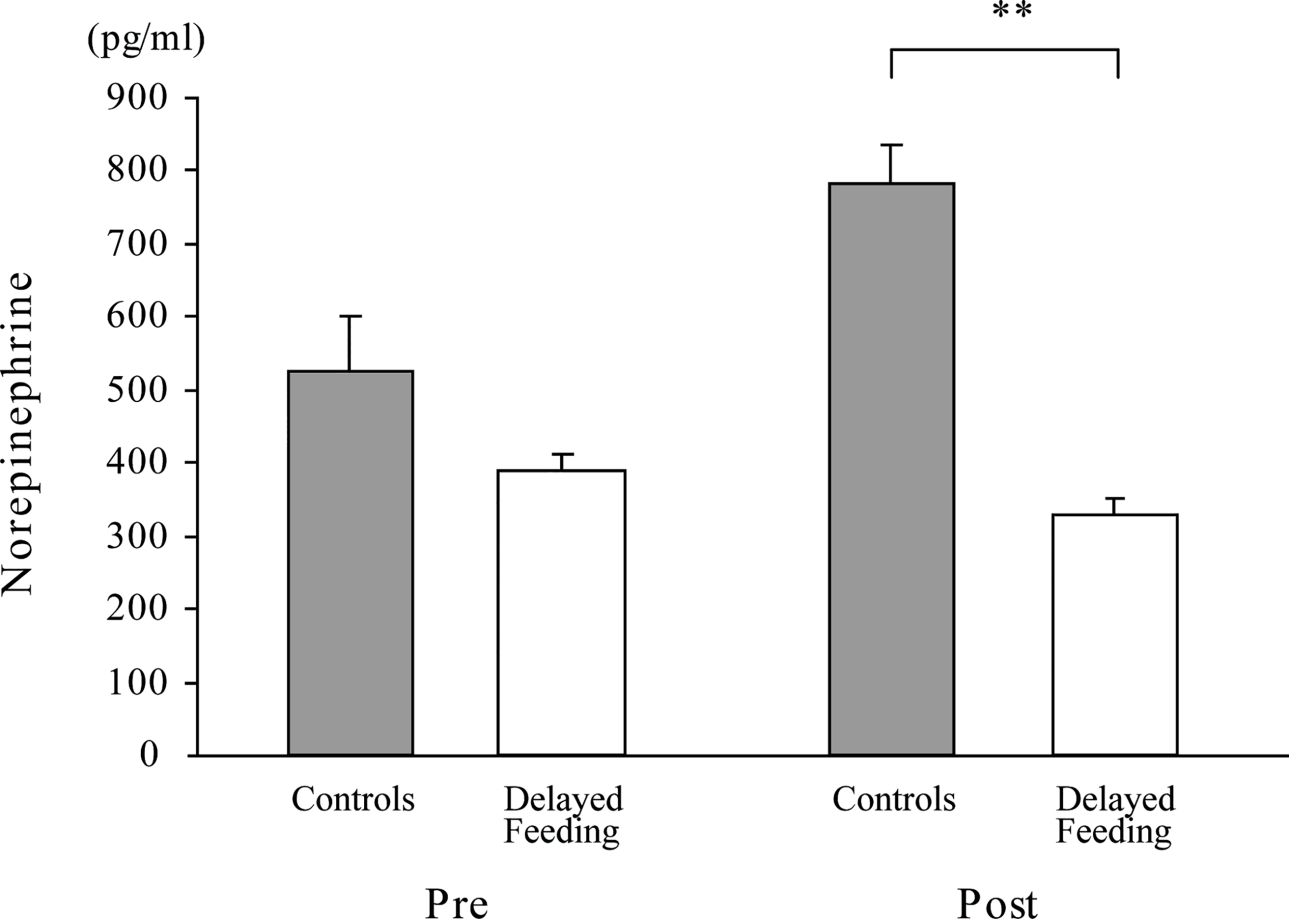
Plasma norepinephrine concentrations pre- and post-feeding of the Fast group of dogs, which were dogs that consumed their food completely and did not chew every mouthful more than once. On the 10^th^ day of the experiment blood samples were collected pre- and post-delayed feeding (Delayed feeding, n = 9). The control samples were collected before and after the usual feeding on the 9^th^ day of the experiment (Controls, n = 10). ** significantly different between controls and Delayed feeding subjects using Welch’s unequal variance test (t = 3.69; *p* < 0.01).

The plasma NE concentrations of the controls, which were measured on day 9 (Fig 2), significantly increased after feeding when compared with the dogs receiving delayed feedings, according to the Welch’s test (Fig 4).

**Fig 5:**
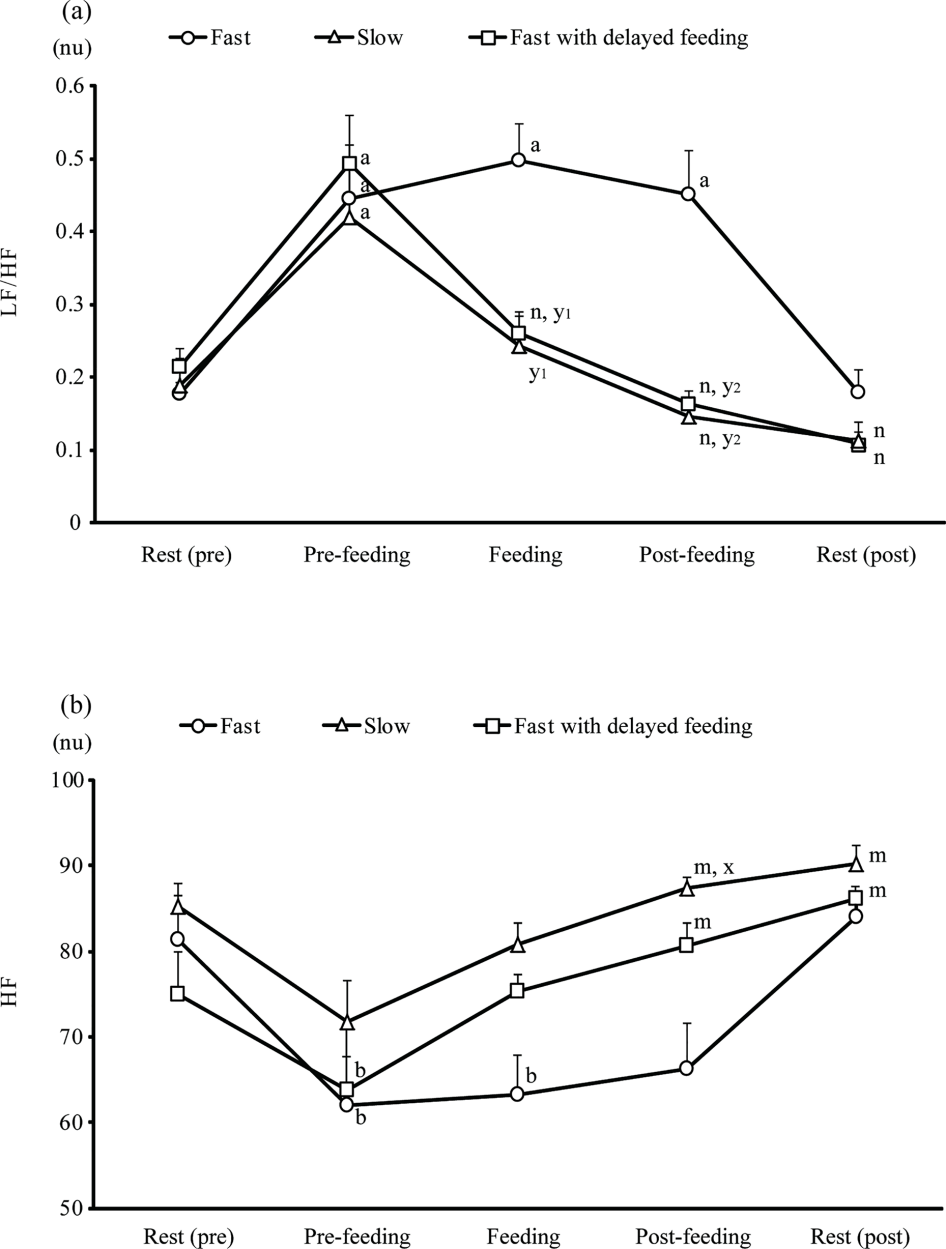
(a) The low frequency/high frequency (LF/HF) ratio and (b) the HF of heart rate variability at rest (Rest (pre)), before (Pre-feeding), during (Feeding), after feeding (Post-feeding) and at rest (Rest (post)) in Fast, Slow, and Fast with delayed feeding, which were fed using automatic feeding equipment, groups of dogs. Fast, Slow and Fast with delayed feeding: See Tables 1 & 2. a) significantly increased, compared with the Rest (pre) (paired t-test, *p* < 0.05 or better); t-values: –3.76, –4.03, and –3.50 at Pre-feeding, Feeding, and Post-feeding, respectively, in Fast; –2.94 at Pre-feeding in Slow; –5.59 at Pre-feeding in Fast with delayed feeding. b) significantly decreased, compared with the Rest (pre) (paired t-test, *p* < 0.05 or better); t-values: 3.56 at Pre-feeding in Fast and 4.81 in Fast with delayed feeding; m) significantly increased, compared with the Pre-feeding (paired t-test, *p* < 0.05 or better); t-values: -3.74 and -4.28 at Post-feeding and Rest (post), respectively, in Slow; -4.81 and 4.74 at Pre-feeding and Rest (post), respectively, in Fast with delayed feeding; n) significantly decreased, compared with the Pre-feeding (paired t-test, *p*<0.05 or better); t-values: 3.14 and 3.44 at Post-feeding and Rest (post), respectively, in Slow; 4.96, 4.82 and 5.45 at Feeding, Post-feeding and Rest (post), respectively, in Fast with delayed feeding; x) significantly increased, compared with the Fast group (Post-feeding) (F(2, 19) = 5.69, *p*<0.01) (Dunnett’s test); y_1_) significantly decreased, compared with the Fast group (Post-feeding) (F(2, 19) = 3.62, *p*<0.05) (Dunnett’s test); y_2_)significantly decreased, compared with the Fast group (Post-feeding) (F(2, 19) = 9.91, *p*<0.01)(Dunnett’s test).

Fig 5 shows the autonomic activities in the Fast (n = 7), Fast with delayed feeding (n = 9) and Slow groups (n = 6). The pre-feeding level of the sympathetic activity and the LF/HF ratio (Fig 5A) were significantly increased compared with those at rest (pre), in all of the groups. The final number of obtained HRV data was less than at the beginning (n = 9 or 10) of the experiment due to technical and/or dog behavior problems. The delayed feeding in the Fast group dogs significantly decreased the LF/HF ratio during feeding and post-feeding, which were almost the same levels as in the Slow group. The HF level (Fig 5B), which is an appropriate parameter of the activity of parasympathetic nervous system, decreased before feeding (Pre-feeding) in the Fast group. The delayed feeding in the Fast group dogs increased the HF levels during post-feeding, which became similar to level of the Slow group.

## Discussion

The three groups, Fast, Slow and Leftover, were defined based on feeding behavior. Some dogs, i.e. the members of the Fast group, had significantly faster feeding speeds, consuming greater amounts of food per mouthful per body weight than other dogs, suggesting that they might not feel satiated. The Fast group also showed significantly higher plasma NE concentrations after feeding. However, the Slow group had a low plasma NE level after feeding and a significantly higher parasympathetic activity (Table 2, Fig 5B).

Feeding in the Fast group may be a result of competitive feeding in their ancestors, the wolves, from which they were domesticated [15,18]. Eating slowly contributes to a lower risk of obesity in humans perhaps because it aids in appetite control. Chewing thoroughly is an effective strategy to reduce the eating rate [26], and some dogs eat food with less chewing [15,18]. In this study, the dogs in the Fast group may have eaten more food with less chewing because they did not chew every mouthful more than once. Thus, they consumed the greatest amount of food (Fig 1A).

The metabolic balance is controlled by the autonomic nervous system, which controls body weight by influencing food intake and energy consumption [27]. Since there is a close relationship between sympathetic activity and food intake, a reduction in the sympathetic response after food intake could induce an increase in the total amount of ingested food [9]. Bray (2000) [9] reviewed several mechanisms concerning the sympathetic nervous system’s effects on feeding behavior. Both serotonin, acting through 5HT1B/2C receptors, and NE, acting through beta2 and/or beta3 receptors, reduce food intake and augment sympathetic activity. The sympathetic activity rises before food intake terminates. This implies that the rise in sympathetic discharge serves as an endogenous satiety signal [6]. These findings suggest that the high levels of plasma NE, which is an indicator of a highly activity sympathetic nervous system, would have decreased with food intake in dogs.

In this study; however, the relationship between the sympathetic activity and the feeding patterns did not agree with these previous reports. The plasma NE concentrations in the Fast group were significantly increased after feeding. The absolute values of the spectra were in the 0.04–0.15 Hz LF and 0.15–1.00 HF ranges [20]. The LF/HF ratio and HF value are used to estimate the sympathetic and parasympathetic activities, respectively [21]. The LF/HF ratios were high in all dog groups prior to feeding (Fig 5A), suggesting that the dogs might be excited due to an increased appetite at pre-feeding [28].

The dog has inherited its eating proclivities and behaviors from its wild relative, the wolf [18,29]. Wolves kill prey sporadically, often going days between feedings [30]. It may be several days before the wolves eat again, and often they must travel far to find food [30]. When food is found, wolves gorge, sometimes eating several kilograms in a single feeding [29,30]. This feed-move-feed regimen appears to have affected their physiological system to regulate satiety. Humans may have utilized the wolves’ eating behavior to train them to obey humans during domestication.

Fasting has been shown to increase the expression of the orexigenic peptides neuropeptide Y (and agouti-related protein in the hypothalamus [31,32]. Moreover, chronic food restriction increased the neuropeptide Y gene’s expression level in the dorsomedial hypothalamus of rats [33]. These proteins are potent hypothalamic orexigenic peptides in humans and animals [34]. These reports suggest that the chronic food restriction in wolves and some dogs has caused a dysfunction in the hypothalamic satiety center.

However, the Slow group presented a high parasympathetic activity, which enhanced the digestive activity after feeding, suggesting the importance of chewing food or eating over an appropriate time span.

## Conclusions

Like wolves, some dogs eat rapidly with less chewing, which resulted in an increased sympathetic activity during feeding, and delayed feeding could be useful for them. Slow eating may activate the parasympathetic nervous system after feeding, which could enhance the activity of the digestive system.
